# Optimizing microbioreactor cultivation strategies for *Trichoderma reesei*: from batch to fed-batch operations

**DOI:** 10.1186/s12934-024-02371-8

**Published:** 2024-04-15

**Authors:** Katja Rohr, Lisa Gremm, Bertram Geinitz, Etienne Jourdier, Wolfgang Wiechert, Fadhel Ben Chaabane, Marco Oldiges

**Affiliations:** 1 Institute of Bio- and Geosciences, IBG-1: Biotechnology, Forschungszentrum Jülich GmbH, Wilhelm-Johnen-Straße, 52428 Jülich, Germany; 2 Institute of Biotechnology, RWTH Aachen University, Worringerweg 3, 52074 Aachen, Germany; 3 IFP Énergies nouvelles, 1 et 4 avenue de Bois-Préau, 92852 Rueil-Malmaison, France; 4 Computational Systems Biotechnology (AVT.CSB), RWTH Aachen University, 52074 Aachen, Germany

**Keywords:** *Trichoderma reesei*, RutC30, filamentous morphology, microbioreactor (BioLector), small-scale cultivation, microscale fed-batch, screening experiments, cellulase production

## Abstract

**Background:**

Filamentous fungi have long been recognized for their exceptional enzyme production capabilities. Among these, *Trichoderma reesei* has emerged as a key producer of various industrially relevant enzymes and is particularly known for the production of cellulases. Despite the availability of advanced gene editing techniques for *T. reesei*, the cultivation and characterization of resulting strain libraries remain challenging, necessitating well-defined and controlled conditions with higher throughput. Small-scale cultivation devices are popular for screening bacterial strain libraries. However, their current use for filamentous fungi is limited due to their complex morphology.

**Results:**

This study addresses this research gap through the development of a batch cultivation protocol using a microbioreactor for cellulase-producing *T. reesei* strains (wild type, RutC30 and RutC30 TR3158) with offline cellulase activity analysis. Additionally, the feasibility of a microscale fed-batch cultivation workflow is explored, crucial for mimicking industrial cellulase production conditions. A batch cultivation protocol was developed and validated using the BioLector microbioreactor, a Round Well Plate, adapted medium and a shaking frequency of 1000 rpm. A strong correlation between scattered light intensity and cell dry weight underscores the reliability of this method in reflecting fungal biomass formation, even in the context of complex fungal morphology. Building on the batch results, a fed-batch strategy was established for *T. reesei* RutC30. Starting with a glucose concentration of 2.5 g l$^{-1}$ in the batch phase, we introduced a dual-purpose lactose feed to induce cellulase production and prevent carbon catabolite repression. Investigating lactose feeding rates from 0.3 to 0.75 g (l h)$^{-1}$, the lowest rate of 0.3 g (l h)$^{-1}$ revealed a threefold increase in cellobiohydrolase and a fivefold increase in $\upbeta$-glucosidase activity compared to batch processes using the same type and amount of carbon sources.

**Conclusion:**

We successfully established a robust microbioreactor batch cultivation protocol for *T. reesei* wild type, RutC30 and RutC30 TR3158, overcoming challenges associated with complex fungal morphologies. The study highlights the effectiveness of microbioreactor workflows in optimizing cellulase production with *T. reesei*, providing a valuable tool for simultaneous assessment of critical bioprocess parameters and facilitating efficient strain screening. The findings underscore the potential of microscale fed-batch strategies for enhancing enzyme production capabilities, revealing insights for future industrial applications in biotechnology.

**Supplementary Information:**

The online version contains supplementary material available at 10.1186/s12934-024-02371-8.

## Introduction

### Fungal enzymes: importance, production and application

Biotechnology has revolutionized various industries by harnessing the power of enzymes produced by microorganisms. The global market for technically relevant enzymes has witnessed remarkable growth, reaching USD 12 billion in 2022 [1, 2]. Moreover, forecasts predict a further rise in this market, projecting it to reach USD 20 billion by 2030 [3]. In 2022, the enzymes market was primarily driven by the production of enzymes by microorganisms, which accounted for 86% of the market revenue [1]. This is due to the numerous advantages of microbial enzyme production: it is fast, cost-effective, scalable and the microorganisms can be genetically engineered [3, 4].

Among the microbial producers, filamentous fungi have been used for their remarkable enzyme production and secretion capabilities for more than a century [5, 6]. Specifically, *Trichoderma reesei*, discovered more than 80 years ago in the Solomon Islands, has gained recognition for its capacity to degrade cellulosic materials [7]. Initially, *T. reesei* enzyme products were of interest primarily for cellulosic ethanol production in the 1970s [8]. However, with the advancement of *T. reesei* as a recombinant host, it has become a valuable source for generating enzymes used in various applications. In 2015, there were approximately 243 commercially available enzymes produced by microbial fermentation, 26 of which used *T. reesei* as a host organism [8]. In particular, *Trichoderma reesei* species are well known for their ability to produce cellulases [9]. Cellulases find application in the food, feed and technical sector, i.a. in the textile industry for the hydrolysis of waste streams to recover glucose and polyester [4].

### Fungal strain and bioprocess development: small-scale cultivation still in its infancy

A wide portfolio of gene editing techniques has been made available for *T. reesei* in recent years [10–12], enabling the rapid generation of numerous strain variants. However, characterizing these strain libraries under well-defined and controlled conditions is difficult to achieve in laboratory-scale stirred tank bioreactors due to their inefficient use of time and resources at the required throughput. Conversely, such characterization is more feasible on a smaller scale, providing valuable results with less financial investment.

Especially in the last decade, small-scale cultivation devices such as the Ambr (Sartorius, Göttingen, DE), BioLector (Beckman Coulter, Brea, US) or bioREACTOR (2mag AG, München, DE) gained popularity due to their ability to provide higher throughput in combination with high process insight and control [13]. For a detailed comparison of the small-scale systems, we refer to the review by Hemmerich et al. (2018). While these systems are well established for bacteria [14], there is a paucity of literature on their use for filamentous fungi. Some first application studies can be found, e.g. by Tamminen et al. using the Ambr 250 [15, 16], Jansen et al. using the BioLector [14, 17, 18] and Bendig and Weuster-Botz using the bioREACTOR 48 [19]. However, these methods are not yet widely employed with filamentous fungi. This research gap can likely be attributed to the complex morphology of filamentous fungi.

One aspect of the complex morphology of filamentous fungi is their distinct life cycle. Growth starts from spores and progresses through germ tube formation and branched hyphal stages to loose mycelium or dense pellets [20]. The mycelial morphology used in industrial *T. reesei* cultivations results in a highly viscous culture suspension. High viscosity reduces the maximum oxygen transfer capacity in stirred tank reactors, potentially causing oxygen limitations unless compensated by increased agitation or gassing [21]. In shaken systems, viscosities exceeding $\simeq 80$ mPa s also decrease the maximum oxygen transfer capacity [21]. In extreme cases, high viscosities can even lead to the ‘out-of-phase’ phenomenon [22].

Overcoming the challenges associated with filamentous morphologies in small-scale cultivation devices is essential for exploiting the advantages of microbioreactors for industrially relevant fungal strains like *T. reesei* RutC30. Therefore, the following research is needed: (i) Development of a batch cultivation protocol using a microbioreactor (BioLector) for three cellulase producing strains of *T. reesei* with offline cellulase activity analysis. (ii) Assessing the feasibility of a microscale fed-batch cultivation workflow. The step from batch to fed-batch operation is particularly crucial, as the industrial production of cellulolytic enzymes requires carbon-limited conditions and is therefore usually operated in fed-batch or continuous mode. Conducting screening experiments solely under batch conditions may therefore not accurately identify the best-performing strain or optimal parameters [14]. We chose the BioLector system for its ability to measure biomass online, allowing real-time monitoring of fungal growth without the need for sampling and offline analysis at distinct points only. Thus, a suitable replacement for insoluble cellulose as an inducer is required to take advantage of online optical measurement capabilities at the microscale. Therefore, as a practical application, the impact of different lactose feeding rates on the cellulase activity of *T. reesei* RutC30 should be evaluated.

## Results and discussion

### Development of batch cultivation protocols

In the development of batch cultivation protocols for three cellulase producing *T. reesei* strains, we investigated two types of BioLector plates, FlowerPlates and Round Well Plates (RWPs). These two plate types offer different well geometries. BioLector FlowerPlates offer a 3 to 4 times higher volumetric oxygen transfer coefficient ($\text{k}_\text{L}$a) than RWPs due to their baffled structure, with $\text{k}_\text{L}$ as ranging from 100 to 650 h^−1^ for FlowerPlates and 30 to 160 h^−1^ for RWPs [23, 24]. In order to introduce sufficient oxygen for fungi growing with a microfilamentous morphology, a high $\text{k}_\text{L}$a is required. Therefore, a FlowerPlate was tested first in the development of a milliliter-scale cultivation workflow.

**Fig. 1:**
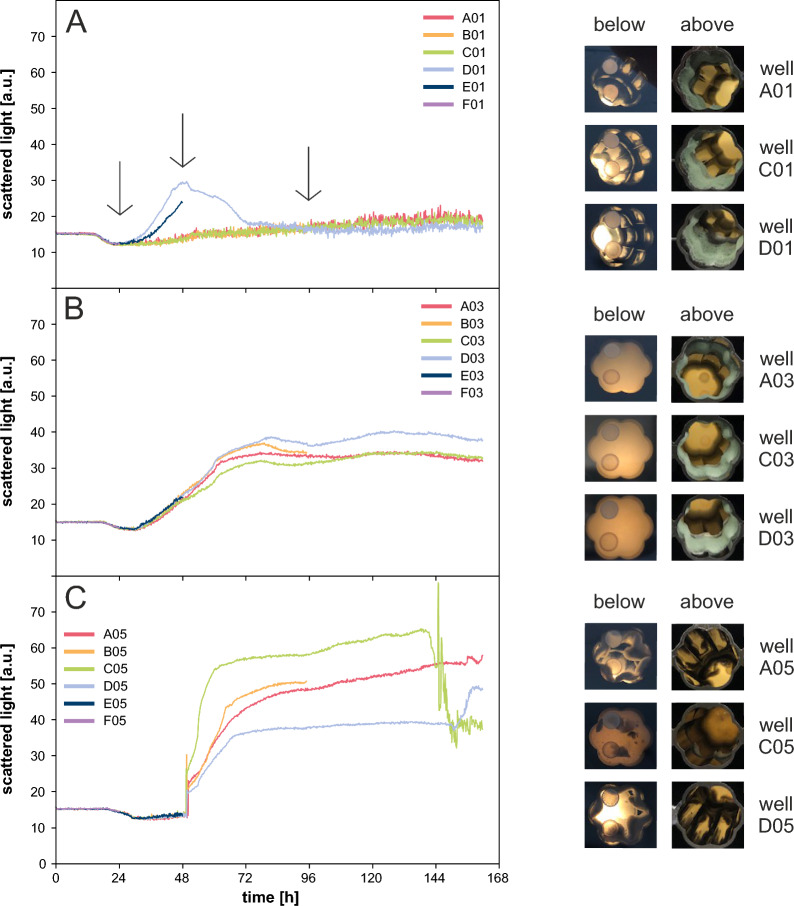
Batch cultivation of *T. reesei* in a FlowerPlate. Scattered light over time and pictures taken from the bottom and top of the cultivation plate at the end of cultivation for **A** wild type, **B** RutC30 and **C** RutC30 TR3158. Reproducibility of the scattered light was low for the wild type and RutC30 TR3158, but high for RutC30. All strains exhibited strong wall growth. Due to this extensive wall growth, the FlowerPlate was considered unsuitable for a reproducible cultivation workflow. Cultivation conditions: FlowerPlate, Jourdier Medium, n = 1200 rpm, ${\text{d}_{0}}=3$ mm, 20 g l$^{-1}$ glucose, ${\text{V}_{\text{W}}}=3.2$ ml, ${\text{V}_\text{L}}$ = 1 ml, humidity $\ge 85$%, ${\text{O}_{2}}$ = 21%, T = 30 $^\circ$C, inoculum = 10$^{5}$ spores ml$^{-1}$, ${\text{n}_\text{bio}}=6$ with sampling of 3 replicates indicated by a black arrow

Figure 1 displays cultivation results of the strains wild type (A), RutC30 (B) and RutC30 TR3158 (C) at a shaking frequency of 1200 rpm and a cultivation volume of 1 ml. For each strain, six biological replicates were run with sampling of three replicates each. The reproducibility of the biomass growth profile measured by scattered light was low for the wild type and RutC30 TR3158, but high for RutC30. A more detailed examination revealed that only two replicates of the wild type showed biomass growth measurable by an increase in scattered light. In contrast, an increase in scattered light was visible for all replicates of RutC30 and RutC30 TR3158. These observations can most likely be attributed to the strong wall growth observed for all strains (Fig. 1, right side). While the wild type and RutC30 adhered to the wall of the well above the cultivation suspension, RutC30 TR3158 formed a thick rim at the bottom of the well. The preferential growth of wild type biomass along the well’s edge rather than in suspension elucidates the absence of a notable increase in scattered light for most replicates. Due to the extensive wall growth and the partly missing growth in suspension, the FlowerPlate was considered unsuitable for a reproducible cultivation workflow. Different cultivation vessel geometries exert different levels of shear stress on the microorganisms in a culture suspension [25]. As shear stress has an important influence on fungal morphology [26–28], this could also affect the wall growth behaviour. Therefore, following cultivations were conducted in RWPs.

RWPs yield a lower $\text{k}_\text{L}$a than FlowerPlates at the same rotational frequency and filling volume [23, 24]. However, the maximum rotational frequency, which is recommended by the manufacturer for RWPs with a filling volume of 1 ml, is 1000 rpm. Therefore, in order to avoid contact between the culture suspension and the sealing foil, the rotational frequency was reduced to 1000 rpm in subsequent cultivations to remain within the manufacturer’s specifications.

**Fig. 2:**
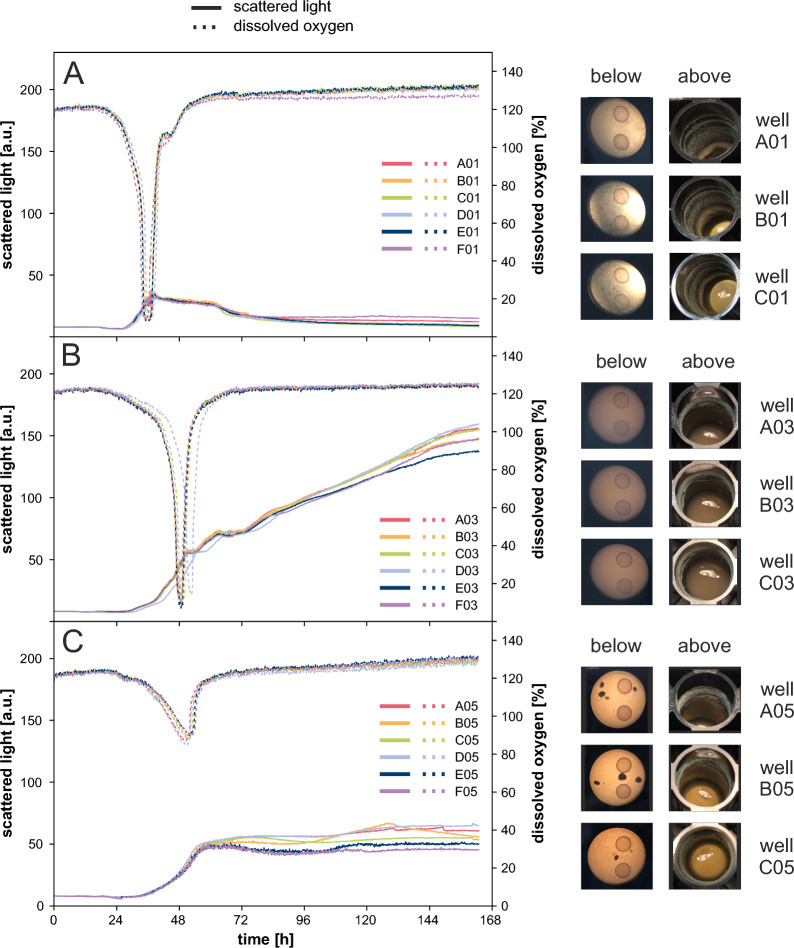
Batch cultivation of *T. reesei* in a RWP at 1000 rpm. Scattered light and DO over time as well as pictures taken from the bottom and top of the cultivation plate at the end of cultivation for **A** wild type, **B** RutC30 and **C** RutC30 TR3158. Scattered light and DO showed high reproducibility between biological replicates for all strains. Compared to the previous cultivations, wall growth was drastically reduced and contact between cultivation suspension and sealing foil was successfully prevented. Cultivation conditions: RWP, Jourdier Medium, n = 1000 rpm, ${\text{d}_{0}}=3$ mm, 20 g l$^{-1}$ glucose, ${\text{V}_{\text{W}}}=3.4$ ml, ${\text{V}_\text{L}}=1$ ml, humidity $\ge 85$%, ${\text{O}_{2}}=21$%, T = 30 $^\circ$C, inoculum = 10$^{5}$ spores .ml$^{-1}$, ${\text{n}_\text{bio}}=6$

Figure 2 shows the results of all three strains cultivated in a RWP at 1000 rpm with six biological replicates each. It illustrates the high reproducibility of the time course of scattered light and dissolved oxygen (DO). This can be exemplified by the mean value and standard deviation of the time at which the DO minimum was reached (not depicted). The DO minimum was reached at t_wt_ = 36.0 ± 0.5 h (Fig. 2A), t_RutC30_ = 49.5 ± 1.4 h (Fig. 2B) and t_RutC30 TR3158_ = 51.4 ± 0.9 h (Fig. 2C). This shows very similar growth profiles between replicates for all three strains based on scattered light and DO signal. Compared to the previous cultivations, wall growth was considerably reduced (compare Figs. 1 and 2, both right side) and contact between cultivation suspension and sealing foil was successfully prevented. However, for wild type and RutC30, the DO signal decreased to approximately 10%, possibly resulting in short-term oxygen limitation in these cultures (Fig. 2A, B). This was not observed in previous cultivations using a FlowerPlate at 1200 rpm or a RWP at 1200 rpm (not depicted). In contrast, the DO of RutC30 TR3158 only decreased to approximately 90%. According to the findings of Giese et al. [21], the maximum oxygen transfer capacity in shake flasks decreases when the viscosity exceeds approximately 80 mPa s. Given that microtiter plates are similar to shake flasks in terms of the geometry of the surface aeration mechanism, it’s likely that higher viscosities also result in lower maximum oxygen transfer capacities. RutC30 TR3158 is a spontaneous mutant derived from RutC30 that results in a lower viscosity of the culture suspension. It is therefore highly likely, that the lower viscosity of the RutC30 TR3158 cultures provided a higher oxygen transfer capacity, thereby avoiding oxygen limitations.

A medium adaptation can potentially increase the maximum oxygen transfer capacity by producing morphologies that lead to a lower viscosity [29]. Moreover, a medium adaptation was required to provide more flexibility in medium preparation and to reduce complex and undefined side reactions during autoclaving. Thus, the medium was adapted for subsequent cultivations as described in section “Cultivation media”. In the cultivation experiments shown so far, the Jourdier Medium was used, which is a complex medium consisting of mineral salts, buffer, trace elements, 1.5 g l$^{-1}$ cornsteep solids and glucose [30]. As described in the literature, the medium including all components is finally autoclaved, which allows the components to react with each other. To avoid this and to increase flexibility, medium preparation was changed to individual stock solutions. These stocks were autoclaved separately and combined immediately before an experiment to form the complete medium used for subsequent cultivations. This medium was labelled “adapted medium”.

**Fig. 3:**
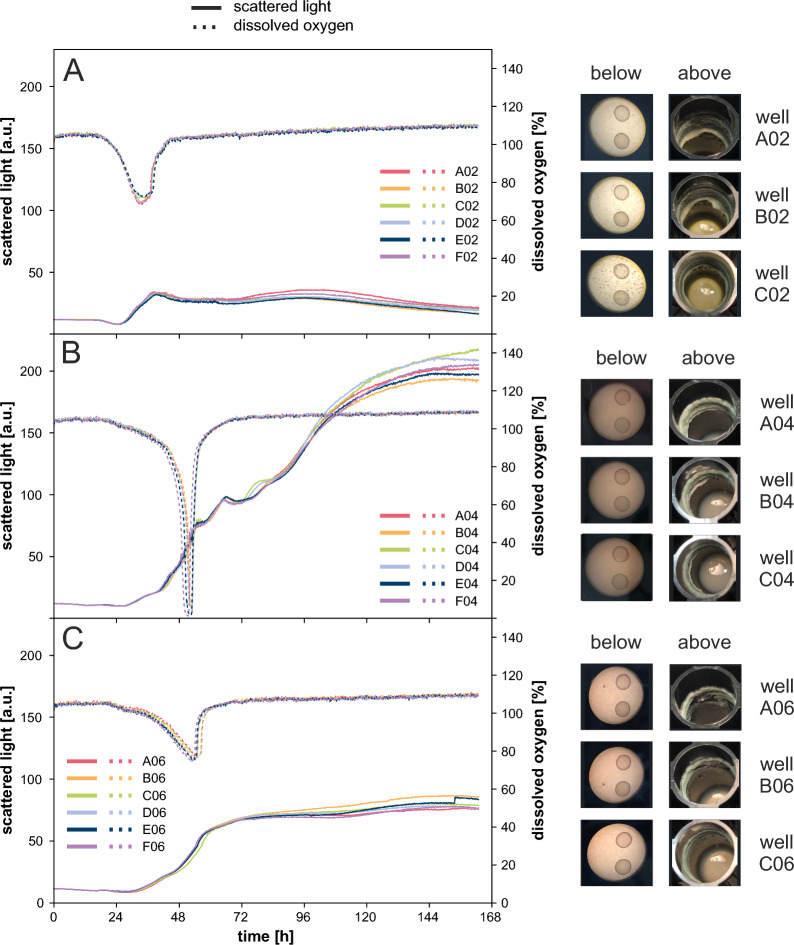
Batch cultivation of *T. reesei* with adapted medium. Scattered light and DO over time as well as pictures taken from the bottom and top of the cultivation plate at the end of cultivation for **A** wild type, **B** RutC30 and **C** RutC30 TR3158. Reproducibility of scattered light and DO for biological replicates was high for all strains in both media. However, cultures with adapted medium showed even higher reproducibility between biological replicates. Wall growth was comparably low for both media. Cultivation conditions: RWP, adapted medium, n = 1000 rpm, ${\text{d}_0}=3$ mm, 20 g l$^{-1}$ glucose, ${\text{V}_\text{W}}=3.4$ ml, ${\text{V}_\text{L}}=1$ ml, humidity $\ge 85$%, ${\text{O}_{2}}=21$%, T = 30 $^\circ$C, inoculum = 10$^{5}$ spores ml$^{-1}$, ${\text{n}_\text{bio}}=6$

Figure 3 depicts cultivations with adapted medium for all three strains. As before, the reproducibility of scattered light and DO can be exemplified by the mean and standard deviation of the time the DO minimum was reached (not depicted). The DO minimum was reached at t_wt_ = 33.9 ± 0.6 h (Fig. 3A), t_RutC30_ = 52.5 ± 0.6 h (Fig. 3B) and t_RutC30 TR3158_ = 53.9 ± 0.9 h (Fig. 3C). This low standard deviation of ${\sigma }\le$ 0.9 h demonstrates an even higher reproducibility of biological replicates in the adapted medium than in the Jourdier Medium (compare Figs. 2 and 3). This higher reproducibility most likely results from the prevention of complex and undefined side reactions during autoclaving. Furthermore, the standard deviations between strains were very similar compared to the standard deviations of the Jourdier medium. As in the previous cultivation, wall growth in the form of biomass formation at the upper well wall above the cultivation suspension was low (compare Figs. 2 and 3, both right side). No biomass aggregation was observed at the bottom border of any of the wells. The growth profile of the biomass, given by the scattered light, was very comparable between the two media.

The DO profiles of RutC30 and RutC30 TR3158 were also very comparable between Jourdier Medium and adapted medium (compare Figs. 2B and C with 3B and C). However, the profile of the wild type exhibited significant changes (compare Figs. 2A and 3A). Most prominently, the DO went down to approximately 70% only, indicating a much lower oxygen demand of the fungi or an improved maximum oxygen transfer capacity in the adapted medium. As stated before, according to the findings of Giese et al. [21], the maximum oxygen transfer capacity in shake flasks decreases when the viscosity exceeds approximately 80 mPa s. This suggests that adapted medium may result in growth of the wild type with altered morphological characteristics, such as shorter hyphal length or reduced branching and thus reduced viscosity.

Strikingly, no similar effect of altered DO profile was observed for RutC30 and RutC30 TR3158. Since RutC30 was derived from the wild type by three rounds of random mutagenesis [31], it is genetically different from the wild type. This offers a possible explanation as to why no changes in the DO limitation were observed in RutC30. Our hypothesis proposes that, due to the genetic differences, the changes made to the medium do not effect this strain in the same way as the wild type. As previously stated, RutC30 TR3158 is a spontaneous mutant derived from RutC30, yielding a lower viscosity of the culture suspension. This property of lower viscosity appears to be present in both the Jourdier Medium and the adapted medium, resulting in a lower DO decrease under both conditions.

**Fig. 4:**
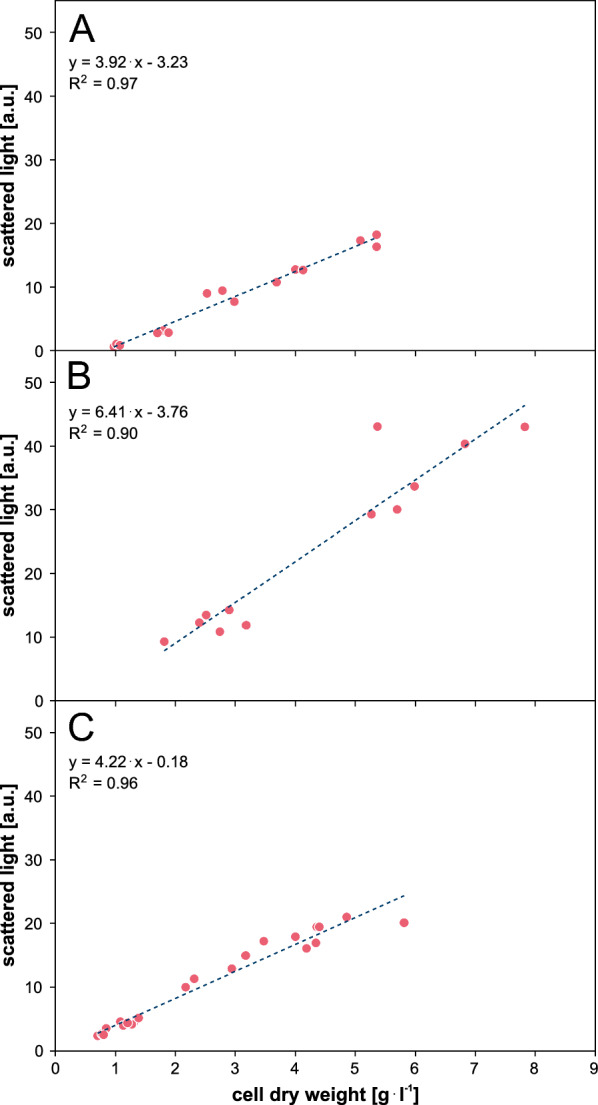
Correlation of scattered light and cell dry weight with a linear regression for **A** wild type, **B** RutC30 and **C** RutC30 TR3158. Regression analysis shows a linear relationship with high coefficients of determination of R^2^_wt_ = 0.97, R^2^_RutC30_ = 0.90 and R^2^_RutC30 TR3158_ = 0.96. This shows that scattered light accurately tracks biomass formation for all three strains during the phase of steep scattered light increase. Cultivation conditions: RWP, adapted medium, n = 1000 rpm, ${\text{d}_0}=3$ mm, 20 g l$^{-1}$ glucose, ${\text{V}_\text{W}}=3.4$ ml, ${\text{V}_\text{L}}=1$ ml, humidity $\ge 85$%, ${\text{O}_{2}}=35$%, T = 30 $^\circ$C, inoculum = 10$^{5}$ spores ml$^{-1}$

The reliable determination of biomass concentration is an important parameter to characterize the cultivation process, e.g. to derive biomass-specific performance indicators. So far, the scattered light of the BioLector online measurement has been used as a representative proxy for biomass concentration. Since scattered light is a complex optical signal, it can be affected by morphological changes during cultivation. Therefore, due to the complex nature of fungal morphology, a linear correlation between scattered light and cell dry weight, as often observed for unicellular microbial systems, cannot necessarily be expected [17]. Figure 4 shows the correlation of scattered light and cell dry weight for all three strains resulting from a cultivation using the developed batch cultivation protocol with adapted medium. Cell dry weight sampling points were chosen throughout the steep increase of scattered light. Surprisingly, the application of regression analysis revealed a linear relationship with high coefficients of determination of R^2^_wt_ = 0.97, R^2^_RutC30_ = 0.90 and R^2^_RutC30 TR3158_ = 0.96. This allows us to conclude that the scattered light accurately tracks biomass formation for all three strains during the phase of steep scattered light increase when the developed batch cultivation protocol is applied. The linear correlation for all three *T. reesei* strains highlights the adaptability and effectiveness of the batch protocol to accommodate different genetic backgrounds. In conclusion, these results highlight scattered light measurement as a non-invasive real-time monitoring technique for *T. reesei* wild type, RutC30 and RutC30 TR3158.

### Applying industrially relevant conditions: fed-batch cultivation

The developed cultivation protocol can be used for the screening of new *T. reesei* strain constructs. However, as stated by Scheidle et al. [32], to ensure the selection of the most promising candidate, it is crucial to closely match the screening conditions with industrial production conditions. Therefore, we evaluated the suitability of a microfluidic fed-batch approach to cellulase production processes using *T. reesei*, which are commonly conducted in fed-batch mode [33].

The fed-batch process was designed using glucose for cell growth in the batch phase and lactose to induce cellulase production in the fed-batch phase. Prior to initiating the fed-batch cultivation, we first needed to determine the appropriate ratio of batch to fed-batch substrate, i.e. glucose to lactose. This was achieved by conducting batch cultivation experiments with varying glucose to lactose ratios. Our aim was to identify the combination that yields the highest cellulase activities. This data can then be used to examine whether these activities can be further improved using the same amount of glucose and lactose, but in a fed-batch mode, to allow a fair comparison of the two process modes. The total carbon source amount was maintained at an equimolar equivalent of 20 g l$^{-1}$ glucose, in alignment with previous batch cultivations.

**Fig. 5:**
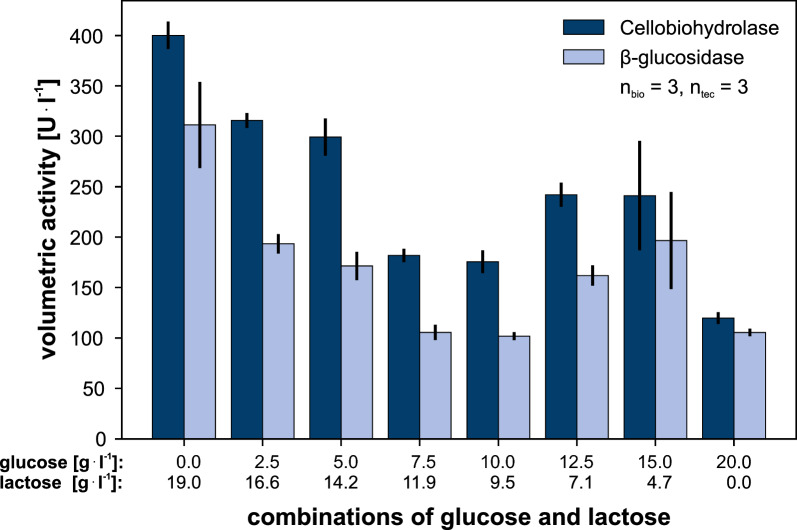
Volumetric activity of cellobiohydrolase and $\upbeta$-glucosidase for different combinations of glucose and lactose. The experiment with 0 g l$^{-1}$ lactose and 20 g l$^{-1}$ glucose resulted in the lowest cellobiohydrolase and $\upbeta$-glucosidase activities, measuring at 120 ± 6 U l$^{-1}$ and 106 ± 4 U l$^{-1}$. The highest cellulase activities of 400 ± 14 U l$^{-1}$ and 311 ± 43 U l$^{-1}$ were achieved when lactose was used as the sole carbon source. This represents a threefold increase in activity compared to the standard batch approach using glucose as the sole carbon source. Cultivation conditions: RutC30, RWP, adapted medium, n = 1000 rpm, ${\text{d}_{0}}=3$ mm, ${\text{V}_\text{W}}=3.4$ ml, ${\text{V}_\text{L}}=1$ ml, humidity $\ge 85$%, ${\text{O}_{2}}=35$%, T = 30 $^\circ$C, inoculum = 10$^{5}$ spores ml$^{-1}$

Figure 5 shows the volumetric activity of two cellulases, cellobiohydrolase and $\upbeta$-glucosidase, produced by RutC30, which play a crucial role in cellulose hydrolysis. Surprisingly, we observed a non-linear relation between the activity of cellulases and the quantity of lactose added. The experiment with 0 g l$^{-1}$ lactose and 20 g l$^{-1}$ glucose resulted in the lowest cellobiohydrolase and $\upbeta$-glucosidase activities, measuring at 120 ± 6 U l$^{-1}$ and 106 ± 4 U l$^{-1}$, respectively. With increasing lactose concentrations of 4.7 and 7.1 g l$^{-1}$, we observed an increase in cellulase activities. These results agreed with our expectations, since lactose acts as an inducer of cellulase production, whereas glucose acts as a repressor [30, 33]. Interestingly, this trend does not continue, but a decrease in cellulase activities was observed at lactose concentrations of 9.5 g l$^{-1}$ and 11.9 g l$^{-1}$. Nevertheless, the highest cellulase activities of 400 ± 14 U l$^{-1}$ and 311 ± 43 U l$^{-1}$ were achieved when lactose was used as the sole carbon source. This represents a threefold increase in activity compared to the standard batch approach using glucose as the sole carbon source. Importantly, in all cases, complete uptake of glucose and lactose was confirmed by a characteristic increase in DO, indicating the full consumption of the primary carbon sources. This data is included in Additional file 1: Figure S1. In order to facilitate a cell growth phase in the fed-batch cultivation, we selected a configuration of 2.5 g l$^{-1}$ glucose followed by 17 g l$^{-1}$ lactose for the fed-batch process, as this resulted in considerable activities for cellobiohydrolase and $\upbeta$-glucosidase as well.

**Fig. 6:**
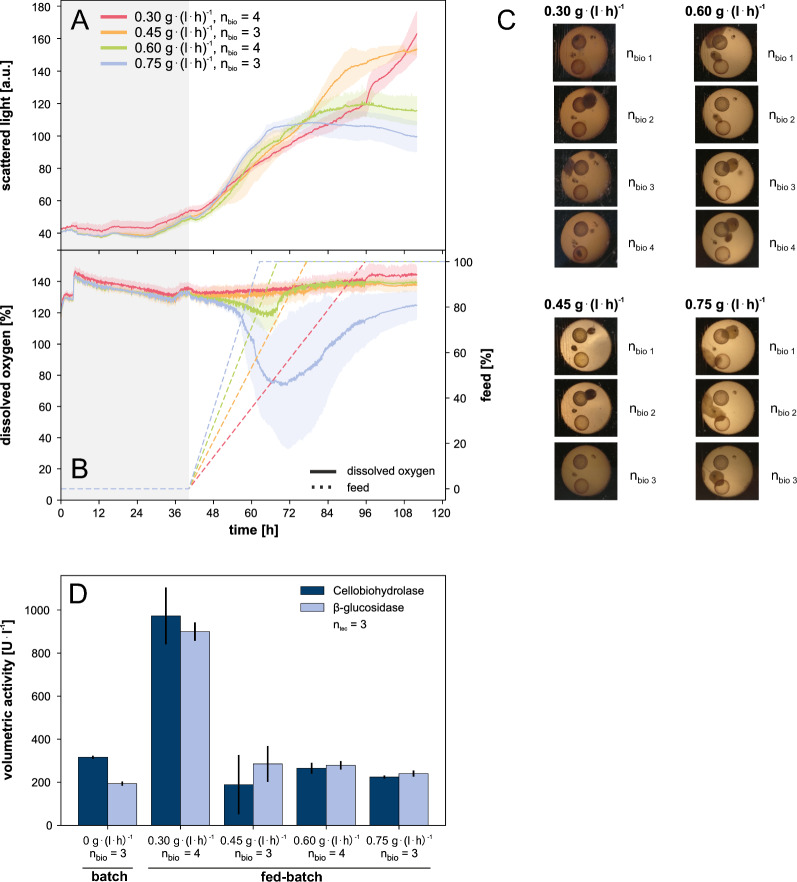
Fed-batch cultivation of *T. reesei* RutC30. **A** Scattered light, **B** DO and feed over time. The line graph presents the mean of the replicates, while the colored areas surrounding the line illustrate the standard deviation. **C** pictures taken from the bottom of the cultivation plate at the end of cultivation, **D** offline measurement of cellulase activities for batch and fed-batch cultivations using the same overall carbon sources (2.5 g l$^{-1}$ glucose and 16.6 g l$^{-1}$ lactose). The DO shows that the feeding rates were appropriately chosen to cover the range from limiting to high substrate feeding. The highest feeding rate of 0.75 g (l h)$^{-1}$, which was overfed based on the DO, led to low cellulase activities, similar to batch cultivations with identical concentrations of glucose and lactose. In contrast, for the feeding rates of 0.3 g (l h)$^{-1}$, 0.45 g (l h)$^{-1}$ and 0.6 g (l h)$^{-1}$, which are assumed to be carbon limited, only the lowest feeding rate of 0.3 g (l h)$^{-1}$ revealed a significant increase in cellulase activities compared to the other conditions. Specifically, there was a threefold increase in cellobiohydrolase and a fivefold increase in $\upbeta$-glucosidase activity compared to the batch conditions. Notably, a morphological change to a combination of microfilamentous structures and pellets was observed in the fed-batch cultivation. Cultivation conditions: Microfluidic RWP, adapted medium, n = 1000 rpm, ${\text{d}_{0}}=3$ mm, ${\text{V}_\text{W}}=3.4$ ml, ${\text{V}_\text{L}}=1$ ml, humidity $\ge 85$%, ${\text{O}}_2=35$%, T = 30 $^\circ$C, inoculum = 10$^{5}$ spores ml$^{-1}$, batch: 2.5 g l$^{-1}$ glucose, fed-batch: 16.6 g l$^{-1}$ lactose as constant feed with a feeding rate of 0.30, 0.45, 0.60 or 0.75 g (l h)$^{-1}$

Figure 6 presents the results of a fed-batch cultivation using the predetermined amount of glucose in the batch phase and lactose as a feed. The experiment investigated the influence of four different feeding rates on metabolic activity and cellulase activity of RutC30. The feeding rates were estimated based on the lactose consumption rate from the previous experiment, targeting a balance between carbon limitation and overfeeding. The start time for feeding was determined using glucose consumption data from preliminary experiments. These experiments revealed that an increase in DO occurs after approximately 40 h in a batch experiment that uses 2.5 g l$^{-1}$ glucose (not depicted).

During the initial batch phase of all 14 fed-batch experiments, the signals of scattered light and DO were very similar between replicates, demonstrating very good comparability. After approximately 40 h, the DO signal indicated the depletion of the glucose batch substrate and the fed-batch phase was started. In the first 75 h of cultivation, there was minimal influence of the different feeding rates on scattered light measurements (Fig. 6A). However, the observation of DO clearly showed that the feeding rates were appropriately selected to cover the range from limiting to high substrate feeding (Fig. 6B). The two lowest feeding rates, 0.3 g (l h)$^{-1}$ and 0.45 g (l h)$^{-1}$, had no effect on the DO levels, indicating a strong carbon limitation. The feeding rate of 0.6 g (l h)$^{-1}$ led to a moderate decrease in DO. However, when the feeding stopped, there was a sudden increase in DO, clearly indicating a carbon limiting feed profile. The highest feeding rate, 0.75 g (l h)$^{-1}$, resulted in a strong decrease in the DO after feed start. Even after the feeding stopped, the DO signal continued to decrease, suggesting a state of strong overfeeding.

Comparing Figs. 3 and 6C, a noticeable morphological change from pure microfilamentous structures in the batch cultivation to a combination of microfilamentous structures and pellets in the fed-batch cultivation was observed. This change could be caused by the use of lactose in addition to glucose as a carbon source. Another possible explanation is the influence of a short starvation phase prior to the initiation of lactose feeding. The activation of the feed occurred approximately 2 h after the initial rise in DO, as depicted in the DO signal Fig. 6B.

The offline measurement of cellulase activities produced surprising results, elucidating the benefits of the optimized fed-batch over the batch process, as Fig. 6D presents. As expected, the highest feeding rate of 0.75 g (l h)$^{-1}$, which was too high for carbon-limited conditions based on the DO signal, resulted in low cellulase activities. These activities were similar to batch cultivations with identical glucose and lactose concentrations. In contrast, at feeding rates of 0.3 g (l h)$^{-1}$, 0.45 g (l h)$^{-1}$ and 0.6 g (l h)$^{-1}$, which were assumed to be carbon limiting, only the lowest feeding rate of 0.3 g (l h)$^{-1}$ revealed a significant increase in cellulase activities compared to the other conditions. Specifically, there was a threefold increase in cellobiohydrolase and a fivefold increase in $\upbeta$-glucosidase activity compared to the batch conditions with identical concentrations of glucose and lactose. The findings strongly suggest that cellulase production is significantly affected not only by the transition to substrate-limiting conditions but also by the degree of carbon limitation. This influence likely operates through the genetic regulation of cellulase production. Remarkably, the lowest feeding rate led to the highest level of enzyme activity. These observations underscore the promise of microbioreactor systems in enhancing enzyme production optimization.

The results highlight the considerable developments in the field of microcultivation through the developed milliliter-scale cultivation workflow and its advantages for fungal bioprocess applications. The high reproducibility of the workflow is demonstrated by the good coherence of replicate cultivation in both batch and fed-batch mode. This workflow enables us to analyze multiple parameters simultaneously, providing a valuable tool for *T. reesei* strain screening, characterization and further investigations. In conclusion, our study has demonstrated the high suitability of implementing a microfluidic fed-batch approach for cellulase production processes employing the fungus *T. reesei*.

## Methods

### Cultivation media

Potato dextrose medium was prepared from 24 g l$^{-1}$ potato dextrose broth and 33 g l$^{-1}$ glucose monohydrate. Potato dextrose agar (PDA) was prepared from 24 g l$^{-1}$ potato dextrose broth and 15 g l$^{-1}$ agar. Both solutions were sterilized by autoclaving at 121 $^\circ$C for 20 min.

For microbioreactor cultivations, two different media were used. The first one, Jourdier Medium, was prepared on the basis of the medium described by Jourdier et al. [30]. This medium consisted of a trace element solution comprising the following components: 0.4 g l$^{-1}$ H$_3$BO$_3$, 9 g l$^{-1}$ Co(NO$_3$)$_2\cdot$6 H$_2$O, 3 g l$^{-1}$ CuSO$_4\cdot$5 H$_2$O, 30 g l$^{-1}$ FeSO$_4\cdot$7 H$_2$O, 6.4 g l$^{-1}$ MnSO$_4\cdot$H$_2$O, 14.3 g l$^{-1}$ 85% H$_3$PO$_4$(aq), 1 g l$^{-1}$ Na$_2$MoO$_4\cdot$2 H$_2$O and 8.4 g l$^{-1}$ ZnSO$_4\cdot$7 H$_2$O. To prepare the final Jourdier Medium, 1 ml l$^{-1}$ trace element solution was combined with the following components: 5.6 g l$^{-1}$ (NH$_4$)$_2$SO$_4$, 6 g l$^{-1}$ 1,2,3,4-butanetetracarboxylic acid, 0.15 g l$^{-1}$ CaCl$_2\cdot$2 H$_2$O, 1.5 g l$^{-1}$ cornsteep solids, 4.4 g l$^{-1}$ K$_2$HPO$_4$, 22 g l$^{-1}$ glucose monohydrate, 0.3 g l$^{-1}$ MgSO$_4\cdot$7 H$_2$O and 4 g l$^{-1}$ KOH. The pH was adjusted to 6.0 by using NaOH and the completed medium was subsequently autoclaved at 110 $^\circ$C for 30 min.

The second medium used for microbioreactor cultivations, adapted medium, had the same chemical composition as the Jourdier Medium, with two exceptions: KOH was excluded and $20\times10^{-3}$ g l$^{-1}$ penicillin G sodium salt was included. However, in the adapted medium, the chemicals were prepared as individual stocks, with each stock’s pH adjusted individually, and sterilized separately. The stocks used were as follows: tenfold buffer stock (60 g l$^{-1}$ 1,2,3,4-butanetetracarboxylic acid), onefold cornsteep solid suspension (1.5 g l$^{-1}$ cornsteep solids), 1000-fold penicillin (20 g l$^{-1}$ penicillin G sodium salt), 15-fold salt stock (84 g l$^{-1}$ (NH$_4$)$_2$SO$_4$, 2.25 g l$^{-1}$ CaCl$_2\cdot$2 H$_2$O, 66 g l$^{-1}$ K$_2$HPO$_4$, 4.5 g l$^{-1}$ MgSO$_4\cdot$7 H$_2$O) and 1000-fold trace element solution (unaltered). Furthermore, for batch and fed-batch experiments, a 400 g l$^{-1}$ glucose stock solution was used to achieve a final concentration of 20 g l$^{-1}$ and 5 g l$^{-1}$, respectively. For fed-batch experiments, a 150 g l$^{-1}$ lactose stock solution was additionally used as the feed. Sterilization was either done by autoclaving at 121 $^\circ$C for 20 min (cornsteep solid suspension, salt stock, glucose stock, lactose stock) or by sterile filtration using a 0.2 $\upmu$m polyethersulfone membrane (buffer stock, penicillin, trace element solution). Immediately before a cultivation, the stocks were combined to form the final medium.

### Strains and strain maintenance

IFP Énergies nouvelles (Rueil-Malmaison, FR) kindly provided the three utilized *T. reesei* strains QM6a (wild type), RutC30 and RutC30 TR3158.

Generation of the main cell banks for QM6a and RutC30 was conducted using the following protocol. The surface of a cryo tube containing a spore suspension was scratched using an inoculation loop. Subsequently, the spore suspension was evenly spread on a PDA plate, which was then incubated at 30 $^\circ$C for a period of 15–20 days, allowing for complete sporulation. Sporulation was indicated by a noticeable green coloration of the biomass. To harvest the spores, 2 ml of a 0.9% (v v$^{-1}$) NaCl solution supplemented with 20% (v v$^{-1}$) glycerol was added to the plate and the surface was scraped using a spatula. Subsequent filtration with a wool filter removed any loose mycelium. We determined the spore concentration using a Neubauer chamber and adjusted it to $10^8$ spores ml$^{-1}$. The resulting spore suspension was aliquoted and stored at − 80 $^\circ$C. Working cell banks were prepared from the master cell bank following the same methodology described above.

For the generation of the main cell bank of RutC30 TR3158, the surface of a cryo tube containing a spore suspension was scratched using an inoculation loop. The spores were then used to inoculate 50 ml of potato dextrose medium in a 250 ml shake flask. It was incubated at 30 $^\circ$C and 250 rpm for a duration of 2 days. After shake flask cultivation, 2 ml of the culture suspension were plated on PDA and further incubated for 15–20 days at 30 $^\circ$C. The subsequent procedure was carried out as described above for the generation of main cell banks of QM6a and RutC30.

### Microbioreactor cultivation

All microbioreactor cultivations were performed in a BioLector Pro (Beckman Coulter, Brea, US). Either FlowerPlates, RWPs or microfluidic RWPs were used, all type BOH1. The plates were sealed with a gas permeable sealing foil. Cultivation conditions were 30 $^\circ$C, 3 mm shaking diameter, $\ge 85$% humidity, 1  ml initial well filling volume, direct inoculation of medium with $10^5$ spores ml$^{-1}$, 1000  rpm or 1200 rpm rotational frequency and 21% or 35% oxygen in the inlet air. During cultivation, scattered light (as a measure of biomass), DO and pH were measured non-invasively in an interval of 10 min. DO and pH were measured using optodes fixed at the bottom of the plate. Unless specifically stated otherwise, 20 g l$^{-1}$ glucose was used as the sole carbon source in batch cultivations. In fed-batch cultivations, the batch phase was performed on 2.5 g l$^{-1}$ glucose and 16.6 g l$^{-1}$ lactose (based on the starting volume) was additionally fed. The choice of lactose concentration was made to ensure it was carbon-equimolar to 15 g l$^{-1}$ glucose, thus providing comparable amounts of carbon source between batch and fed-batch mode.

### Cell dry weight and correlation with scattered light

Cell dry weight was estimated using a pre-weighed Spin-X centrifuge tube filter with a 0.2 $\upmu$m nylon membrane. The filter was loaded with a total of 0.7 ml cell suspension in two steps and centrifuged at 13,000 rcf for 3 min. The retentate was washed twice with 0.5 ml 0.9% NaCl. Washed filters were dried at 80 $^\circ$C for 24 h and stored in a desiccator for 1 h before weighing. Then, the mass difference between the filter containing the retentate and the empty filter was calculated. The difference in mass divided by the volume of cell suspension applied yielded the cell dry weight.

To asses the correlation between cell dry weight and scattered light, all three strains were cultivated using the developed batch protocol as stated in section “Microbioreactor cultivation” with RWPs, 1000 rpm and 35% oxygen. Samples of 0.7 ml each were collected as biological triplicates at various times during the initial steep rise in scattered light. Cell dry weight and scattered light (raw value measured by the BioLector) of each sample were determined. Cell dry weight of each sample was then plotted over the scattered light at the time of sampling. Finally, a linear regression was performed.

### Glucose assay

Glucose analysis was performed using a Glucose Hexokinase FS test kit from DiaSys (Holzheim, DE) according to the manufacturer’s instructions.

### Cellulase activity measurement

For cellulase activity analysis, cultivation samples were filtered using a 0.22 $\upmu$m syringe filter with a polyethersulfone membrane. The supernatant was stored at 4 $^\circ$C for a maximum of 7 days and diluted in citrate buffer if necessary. The citrate buffer consisted of 23 mM citric acid monohydrate, 27 mM tri-sodium citrate dihydrate and the pH was adjusted to 4.8.

The volumetric activity of two different classes of cellulolytic enzymes was determined by an automated optical assay on the robotic platform FREEDOM EVO 200 (Tecan, Männedorf, CH). Cellobiohydrolase activity was determined vicariously by Cel7A (cellobiohydrolase I), whereas $\upbeta$-glucosidase activity was determined directly. Cellobiohydrolase I converts 4-nitrophenyl-$\upbeta$-d-lactopyranoside to $\upbeta$-d-lactopyranoside and 4-nitrophenol (pNP), while $\upbeta$-glucosidase converts 4-nitrophenyl-$\upbeta$-d-glucopyranoside to $\upbeta$-d-glycopyranoside and pNP. The resulting product pNP can be quantified by absorbance measurement at 410 nm.

For cellobiohydrolase I activity determination, 50 $\upmu$l of pNPL solution were mixed with 50 $\upmu$l of sample supernatant. For $\upbeta$-glucosidase activity determination, the ratio was 90 $\upmu$l pNPG solution to 10 $\upmu$l sample supernatant. Both mixtures were incubated at 50 $^\circ$C for 30 min. Then, 100 $\upmu$l sodium carbonate solution (189 mM sodium carbonate) was added and the amount of released pNP was detected by measuring absorbance at 410 nm. Cellulase activity of samples was subsequently calculated using a pNP calibration line.

To generate the calibration line, different concentrations of pNP (0.02–5 mM) in citrate buffer were mixed with equal volumes of sodium carbonate solution. The absorbance was then measured at 410 nm. A linear regression was performed on the data set to determine the slope and intercept of the calibration line, which were subsequently used to calculate the concentration of released pNP in the samples (Eq. 1). This concentration enabled the calculation of the volumetric enzyme activity, defined as the amount of enzyme required to release 1 μmol of pNP per minute (Eqs. 2 and 3).

1 $$\begin{aligned}{} & {} \text { Concentration released pNP } \left[ \frac{\upmu \text {mol}}{\text {l}} \right] = \frac{\text {absorbance A [a.u.] - intercept m [a.u.]}}{\text {slope n } \left[ \frac{\text {l}}{\upmu \text {mol}} \right] } \end{aligned}$$

2 $$\begin{aligned}{} & {} \quad \text {Activity [U] } = \frac{\text { released pNP [}\upmu \text {mol]}}{\text {t [min]}} \end{aligned}$$

3 $$Volumetric{\text{ }}activity\left[ {\frac{U}{l}} \right] = \frac{{concentration{\text{ }}released{\text{ }}pNP\left[ {\frac{{\mu mol}}{l}} \right] \cdot dilution}}{{t[\min ]}}$$

### Cultivation data processing and display

All data parsing, processing and plotting were done using python 3.9.7, bletl 1.3.1 [34, 35], matplotlib 3.5.1 [36, 37], numpy 1.21.5 [38], pandas 1.4.1 [39, 40] and seaborn 0.11.2 [41, 42]. In batch cultivations, single replicates were plotted, while plots of fed-batch cultivations show mean and standard deviation of all replicates of one condition. After wells were sampled, their measurements were no longer included in the figures or associated calculations.

Prior to plotting, scattered light, DO and pH values were blanked by determining the mean measured value among all replicates of one condition at cycle 3. The differences between the mean values obtained beforehand and the scattered light, DO and pH values of each replicate measured at cycle 3 were calculated. These differences were than added to every measured scattered light, DO and pH value of the respective replicate. For fed-batch cultivations, scattered light was additionally corrected for the dilution caused by feeding. This was done by multiplying the measured scattered light with the quotient of current total filling volume of the well and well volume at the beginning of cultivation.

## Conclusion

Our study established a robust microbioreactor batch cultivation protocol for three *T. reesei* strains, optimizing key parameters such as microtiter plate geometry and medium composition. A significant finding was the strong correlation between scattered light intensity and cell dry weight during the growth phase, highlighting scattered light as a reliable biomass indicator in batch cultivation. This is particularly noteworthy given the complex nature of fungal morphology in comparison to unicellular microbial systems. However, the application of this correlation to fed-batch cultivations remains to be explored.

The fed-batch strategy for *T. reesei* RutC30, using four different lactose feeding rates, increased cellobiohydrolase activity threefold and $\upbeta$-glucosidase activity fivefold. These results highlight the potential of microbioreactor systems to optimize enzyme production. Despite these advances, the applicability of our findings to other strains or filamentous organisms remains cautious due to the inherent complexity of fungal morphologies. Future work should aim to validate the correlation of scattered light and cell dry weight in fed-batch processes and extend the microbioreactor protocols to a wider range of fungal strains. These efforts will further elucidate the robustness of the developed protocol and broaden the application of microbioreactors in filamentous fungal research and industrial processes.

### Supplementary Information

**Additional file 1: Fig. S1.** Dissolved oxygen of batch cultivations of T. reesei RutC30 using different glucose and lactose combinations. Cultivation conditions: RutC30, RWP, adapted medium, n = 1000 rpm, d_0_ = 3 mm, glucose and lactose concentrations as declared in the subplots, V_W_ = 3.4 ml, V_L_ = 1 ml, humidity ≥ 85%, O_2_ = 35%, T = 30 °C, inoculum = 10^5^ spores ml^-1^, n_bio_ = 3.

## Data Availability

The cultivation datasets used in this study are available upon reasonable request from the corresponding author.

## Abbreviations

DO: Dissolved oxygen
$\text{k}_\text{L}\text{a}$: Volumetric oxygen transfer coefficient [$\text{h}^{-1}$]
PDA: Potato dextrose agar
pNP: 4-Nitrophenol
RWP: Round well plate
$$\sigma$$: Standard deviation [–]
