# Supplementary material for: Optimizing microbioreactor cultivation strategies for Trichoderma reesei: from batch to fed-batch operations
Source: Microb Cell Fact. 2024 Apr 15;23:112. doi: 10.1186/s12934-024-02371-8 (PMC11334512; doi:10.1186/s12934-024-02371-8)
Supplement: Supplementary file 1 — Additional file 1: Fig. S1. Dissolved oxygen of batch cultivations of T. reesei RutC30 using different glucose and lactose combinations. Cultivation conditions: RutC30, RWP, adapted medium, n = 1000 rpm, d0 = 3 mm, glucose and lactose concentrations as declared in the subplots, VW = 3.4 ml, VL = 1 ml, humidity ≥ 85%, O2 = 35%, T = 30 °C, inoculum = 105 spores ml-1, nbio = 3. [file 12934_2024_2371_MOESM1_ESM.pdf]

Optimizing microbioreactor cultivation strategies for *Trichoderma reesei*: from batch to fed-batch operations

Katja Rohr[1,2], Lisa Gremm[1,2], Bertram Geinitz[1], Etienne Jourdier[3], Wolfgang Wiechert[1,4], Fadhel Ben Chaabane[3], Marco Oldiges[1,2*]

[1*]Institute of Bio- and Geosciences, IBG-1: Biotechnology, Forschungszentrum Jülich GmbH, Wilhelm-Johnen-Straße, Jülich, 52428, Germany.

[2]Institute of Biotechnology, RWTH Aachen University, Worringerweg 3, Aachen, 52074, Germany.

[3]IFP Énergies nouvelles, 1 et 4 avenue de Bois-Préau, Rueil-Malmaison, 92852, France.

[4]Computational Systems Biotechnology (AVT.CSB), RWTH Aachen University, Aachen, 52074, Germany.

*Corresponding author(s). E-mail(s): m.oldiges@fz-juelich.de; Contributing authors: k.rohr@fz-juelich.de; lisa.gremm@rwth-aachen.de; b.geinitz@fz-juelich.de; etienne.jourdier@ifpen.fr; w.wiechert@fz-juelich.de; fadhel.ben-chaabane@ifpen.fr;

Additional information

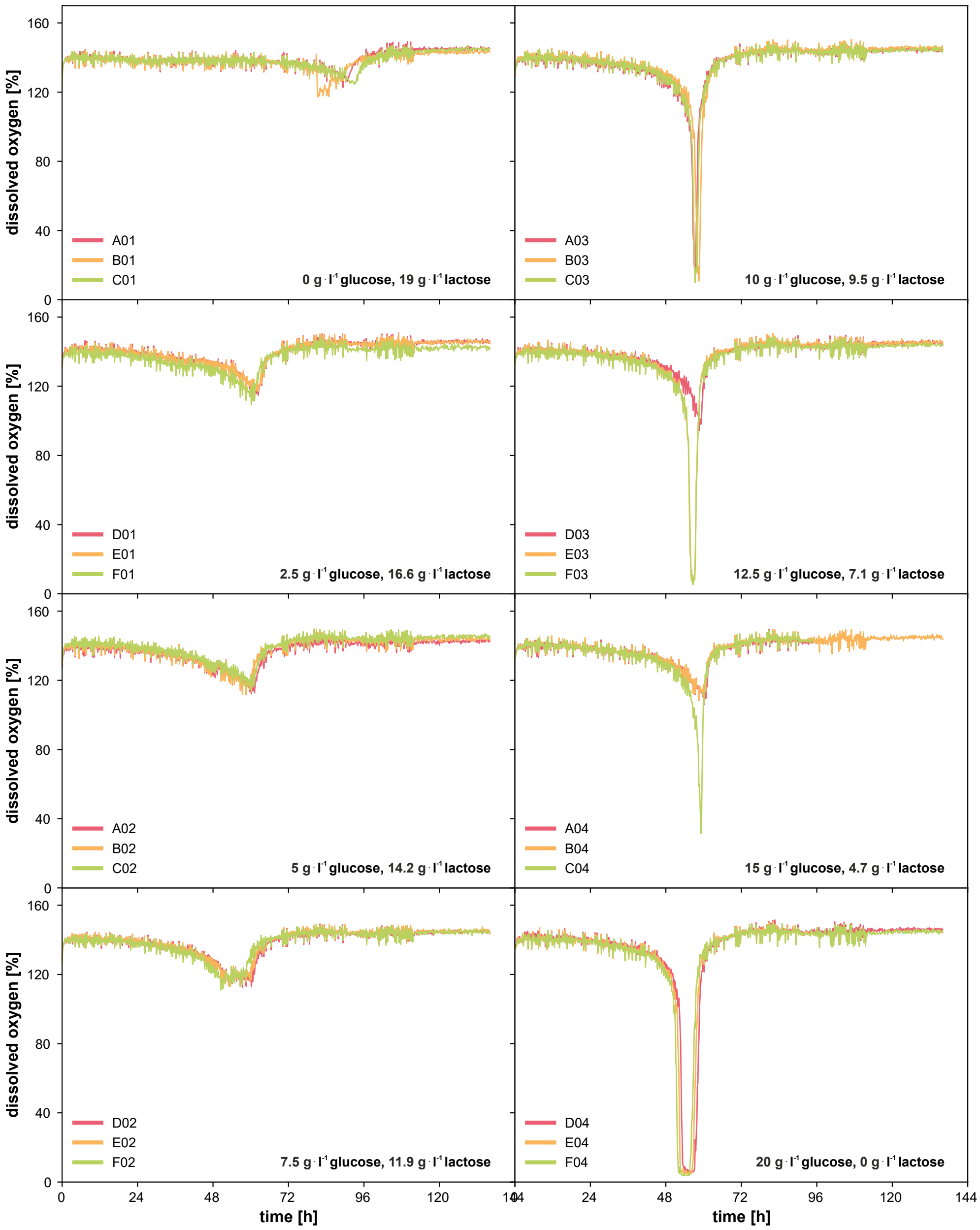

Fig. S1 Dissolved oxygen of batch cultivations of *T. reesei* RutC30 using different glucose and lactose combinations. Cultivation conditions: RutC30, RWP, adapted medium, n = 1000 rpm, d_0 = 3 mm, glucose and lactose concentrations as declared in the subplots, V_W = 3.4 ml, V_L = 1 ml, humidity $\geq$ 85 %, O_2 = 35 %, T = 30 °C, inoculum = 10^5 spores·ml^{-1}, n_{bio} = 3
